# Strategies to Reduce Left Anterior Descending Artery and Left Ventricle Organ Doses in Radiotherapy Planning for Left-Sided Breast Cancer

**DOI:** 10.31083/RCM26366

**Published:** 2025-02-21

**Authors:** Umut Diremsizoglu, Nezihan Topal, Aykut Oguz Konuk, Ibrahim Halil Suyusal, Dogacan Genc, Onur Ari, Hasan Furkan Cevik, Aysegul Ucuncu Kefeli, Maksut Gorkem Aksu, Emine Binnaz Sarper

**Affiliations:** ^1^Department of Radiation Oncology, School of Medicine, Kocaeli University, 41001 Kocaeli, Turkey

**Keywords:** left breast cancer, left anterior descending artery, left ventricle, major adverse cardiac events

## Abstract

**Background:**

One of the most significant long-term toxicities of breast cancer radiotherapy is major adverse cardiac events (MACE). In current radiotherapy practice, the mean heart dose is the most commonly used parameter. The aim of our study was to reduce the doses of organs at risk (OAR) in the left anterior descending artery (LAD) and left ventricle (LV) by including the LAD and LV in planning radiotherapy while maintaining adequate dose coverage for patients with left-sided breast cancer.

**Methods:**

We retrospectively analyzed left-sided breast cancer cases treated at the Kocaeli University Faculty of Medicine. Only patients with local and locally advanced breast cancer were included in the analysis. A total of 77 patients who were treated between 2020 and 2024 were included. The doses to the LAD and LV were added to the optimization algorithms. Two volumetric modulated arc therapy (VMAT) plans were created for each patient. A total of 154 plans were made, including standard and LAD and LV sparing plans.

**Results:**

There was no statistically significant difference in all VMAT plans regarding planning target volume (PTV) D2, D50, and D98 (dose receiving volume of PTV 2%, 50%, and 98%) (*p* > 0.05). However, a significant decrease was observed in heart V5 (the percentage of the heart receiving at least 5 gray (Gy)) and mean heart dose. A decrease in the mean heart dose was observed in the standard plan compared with the LAD and LV sparing plan (*p* < 0.001). Similarly, the heart V5 value decreased significantly (*p* < 0.001). Additionally, significant reductions were measured in all LAD and LV parameters after re-optimization.

**Conclusions:**

We achieved significant reductions in all heart, LAD, and LV parameters without making any changes to the planned treatment volume coverage by adding LAD and LV OARs to the optimization algorithms. The potential risk of MACE can be significantly reduced by implementing this strategy.

## 1. Introduction

The World Health Organization reports that breast cancer is one of the most common cancers worldwide, with approximately 2.3 million new diagnoses annually [1]. The majority of recently diagnosed patients are treated with radiotherapy as adjuvant therapy to breast-conserving surgery [2]. Radiotherapy is associated with longer overall survival and longer local progression-free survival [3]. Radiotherapy is also used in palliative settings to enhance patients’ quality of life.

Due to anatomical proximity and the limitations of current techniques, the spread of radiation to normal tissues is inevitable. The most frequent acute toxicity in breast cancer radiotherapy is skin toxicity, whereas major adverse cardiac events (MACE) are late toxicities. The RTOG0617 study showed that in locally advanced non-small cell lung cancer (NSCLC) patients, overall survival is related to heart V5 and V30 volumes [4]. In current radiotherapy practice, the heart is generally contoured as a single organ and the mean heart dose (MHD) is the most commonly used organ at risk (OAR). According to a study by Darby *et al*. [5], an increase of 1 gray (Gy) in MHD was associated with a 7.4% increase in the risk for MACE. To mitigate MHD and the MACE risk, deep inspiration breath hold (DIBH) is used [6]. DIBH can also be used to reduce the doses to cardiac structures, especially left anterior descending artery (LAD) [7]. In 2011, Vikström *et al*. [8] showed that the MHD and LAD can be reduced by utilizing the DIBH method by as much as 54% and 65%, respectively. A study by Tang *et al*. [9] has shown significant correlations between MHD and doses received by the left ventricular (LV) and right ventricular (RV) anterior and apical walls. Tjong *et al*. [10] found that the key factors in predicting MACE risk after radiotherapy are pre-existing hypertension, coronary heart disease, and LAD V15 (LAD volume receiving 15 Gy in standard plan). The Cardiac disease, Hypertension, and Logarithmic Left anterior descending coronary artery radiation dose (CHyLL) score incorporates LAD V15 rather than MHD to calculate personalized LAD V15 constraints based on cardiac risk factors [10].

This study aimed to dosimetrically reduce LAD and LV doses as OARs by including them in planning optimization for patients with left-sided breast cancer.

## 2. Materials and Methods

We retrospectively analyzed cases of left breast cancer treated at the Kocaeli University Faculty of Medicine. In this analysis, only patients with local and locally advanced disease were included. These patients were expected to have a target volume near the heart. Patients who underwent free-breathing (FB) or DIBH computed tomography (CT) scans were included. Exclusion criteria was a predated second cancer diagnosis. All patients who met the inclusion criteria were enrolled in this study.

Institutional review board approval was obtained for this study. The Non-Interventional Clinical Research Ethics Committee of Kocaeli University approved the study, which convened on 14.02.2022 and assigned protocol number 2022/106.

In standard radiotherapy, the heart is contoured and evaluated as a single organ. In this study, the LAD and LV were contoured as substructures and incorporated into the optimization algorithms to minimize the doses while maintaining target volume coverage and adhering to dose constraints for other critical thoracic OARs. An overview of the optimization parameters for both the standard and sparing plans is shown in Table 1. The planning target volume (PTV) and OAR parameters in the standard plan were left unchanged, and only the LAD and LV parameters were added to the optimization algorithm.

**Table 1.** **Optimization parameters for standard and sparing plans**.

Standard plan	Sparing plan
PTV	PTV
Heart	Heart
Contralateral breast	Contralateral breast
Left lung	Left lung
PRV	PRV
	Left anterior descending artery
	Left ventricle

PTV, planned target volume; PRV, planned risk volume.

DIBH and FB CT images were contoured by a radiation oncology specialist according to the Radiation Therapy Oncology Group (RTOG) contouring atlases [11]. The contoured organs included the right and left breasts, lungs, heart, and intracardiac structures, specifically the left anterior descending artery and left ventricle. An experienced medical physicist designed new cardiac-optimized volumetric modulated arc therapy (VMAT) plans by integrating the previously mentioned structures into the Varian (Palo Alto, CA, USA) Eclipse V13.6 treatment planning system plan optimizer. Integration and planning were standardized, and treatment plans for all patients were calculated using the same optimization parameters. The goal was to maximize the protection of intracardiac substructures while ensuring PTV coverage and following dose constraints for OARs.

VMAT plans were developed for all patients. This involved two arcs. The first arc started at 293° with a collimator angle of 30° and was a clockwise arc of 240°. The second arc started at 173° with a collimator angle of 330° and was a 240° counterclockwise arc. Subsequently, the LAD and LV sparing plans (LADLVSP) aimed to spare the LAD and LV. The treatment plans were normalized to ensure that 95% of the PTV would receive a dose of 50 Gy.

Dosimetric data for the conventional and LADLVSP VMAT plans were collected using the Varian (Palo Alto, CA, USA) Eclipse V13.6 planning software. A comparison was made between the standard and LADLVSP plans. V15 is the percentage of volume receiving 15 Gy, and D2 is the maximum dose received to 2% of the PTV. D50 is the median dose received by 50% of the PTV. D98 is the minimum dose applied to 98% of the PTV [12]. Dosimetric data were obtained using the dose-volume histogram. Mean heart dose, heart V5, and heart V30 were used to compare differences in heart doses between plans. PTV D2, D98, and D50 were used to compare dose distribution. Mean LAD dose, LAD D2, and LAD V15 were used to compare LAD doses. Mean LV dose, LV D2, and LV V23 were used to compare LV doses.

Dosimetric data were analyzed for normal distribution using Kolmogrov-Smirnov Test. Non-parametric data were compared between the conventional and LADLVSP groups using a Wilcoxon‘s test with Bonferroni correction. The parametric data were compared using the paired *t*-test. The significance was assessed at the α = 0.05 level. Statistical analyses were conducted using SPSS version 25.0 (SPSS Inc., Chicago, IL, USA).

## 3. Results

77 patients treated from 2020 to 2024 met the inclusion criteria. Table 2 illustrates the demographic and clinical characteristics of the patients. 77 Standard and 77 LADLVSP plans were created for each patient. A total of 154 plans were analyzed. Re-optimized plans met the prescribed treatment dose, while critical organ doses remained within safety limits according to guidelines [13]. Table 3 summarizes the mean changes in the LAD, LV, PTV, and heart in both VMAT plans. No statistically significant differences were found in the VMAT plans for PTV D2, D98, and D50 (*p* > 0.05). However, a significant reduction was seen in heart V5 and mean values. The mean heart dose, which was 6.65 ± 0.15 in the standard plan, decreased to 6.03 ± 0.10 in the LADLVSP (*p* < 0.001). Similarly, the heart V5 percentage, which was 50.8 ± 16.63, decreased to 45.41 ± 13.60 (*p* < 0.001). Significant reductions were also measured for all LAD and LV parameters after re-optimization.

**Table 2.** **Demographic and characteristic distribution of patients**.

		Age group (0–39)	Age group (40–69)	Age group (70+)	Total
Age					56 (31–80)
Stage					
	1a	1	36	10	47
	1b	0	1	0	1
	2a	2	15	1	18
	2b	0	10	0	10
	3a	1	0	0	1
Size					
	≤5 cm	4	59	11	74
	>5 cm	0	3	0	3
ER status					
	Negative	2	11	2	15
	Positive	2	51	9	62
Her-2 status					
	Negative	4	62	11	77
	Positive	0	0	0	0
Tumor quadrant					
	LI (Lower inner)	0	19	4	23
	LO (Lower outer)	2	3	0	5
	UO (Upper outer)	2	26	3	31
	UI (Upper inner)	0	14	4	18
Grade					
	1	1	14	5	20
	2	2	31	5	38
	3	1	17	1	19

According to AJCC Cancer Staging Manual, Eighth Edition, the letters a and b are used to further subdivide a stage based on tumor characteristics. For instance, stage 1a typically indicates a small tumor with no or minimal lymph node involvement, whereas stage 1b may denote similar tumor size but with microscopic nodal involvement. Similarly, the subdivisions in stage 2 (2a vs 2b) reflect differences in tumor size or extent of nodal involvement. Abbreviations: ER, estrogen receptor.

**Table 3.** **Dosimetric comparison of the VMAT plans**.

	Standard plan	LADLVSP	Difference	*p* value
PTV D2 (Gy)	55.69 ± 0.07	55.68 ± 0.07	0.01	*p* = 0.773
PTV D98 (Gy)	48.83 ± 0.02	48.82 ± 0.02	0.01	*p* = 0.464
PTV D50 (Gy)	53.21 ± 0.02	53.21 ± 0.02	0.00	*p* = 0.797
Heart mean (Gy)	6.65 ± 0.15	6.03 ± 0.10	0.62	*p * < 0.001
Heart V5 (%)	50.8 ± 16.63	45.41 ± 13.60	5.40	*p * < 0.001
Heart V30 (%)	0.54 ± 0.08	0.20 ± 0.03	0.34	*p * < 0.001
Lad mean (Gy)	17.32 ± 0.55	12.41 ± 0.52	4.91	*p * < 0.001
Lad D2 (Gy)	29.52 ± 7.14	24.26 ± 7.73	5.26	*p * < 0.001
Lad V15 (%)	51.89 ± 22.19	29.59 ± 21.69	22.30	*p * < 0.001
LV mean (Gy)	7.46 ± 0.18	5.59 ± 0.12	1.87	*p * < 0.001
LV D2 (Gy)	22.14 ± 5.74	17.28 ± 5.32	4.86	*p * < 0.001
LV V5 (%)	58.59 ± 2.23	37.17 ± 2.17	21.42	*p * < 0.001
LV V23 (%)	2.46 ± 2.69	0.91 ± 1.14	1.55	*p * < 0.001
RV V20 (%)	1.28 ± 2.86	0.82 ± 1.92	0.46	*p* = 0.056
Right breast mean (cGy)	381.25	395.62	14.37	*p * < 0.001
Left lung V20 (%)	21.22	19.92	1.30	*p * < 0.001
Right lung V5 (%)	36.29	37.19	0.90	*p* = 0.316

Abbreviations: LADLVSP, left anterior descending coronary artery and left ventricle–sparing plan; PTV, planning target volume; Vn, percentage of the volume receiving n Gy; D2, D50, and D98, dose receiving volume of PTV 2%, 50%, and 98%, respectively; LAD, left anterior descending artery; LV, left ventricle; LAD V15, LAD volume receiving 15 Gy in standard plan; cGy, centigray; Gy, gray; VMAT, volumetric modulated arc therapy; RV, right ventricular. Data are presented as mean ± standard error.

**Fig. 1.**
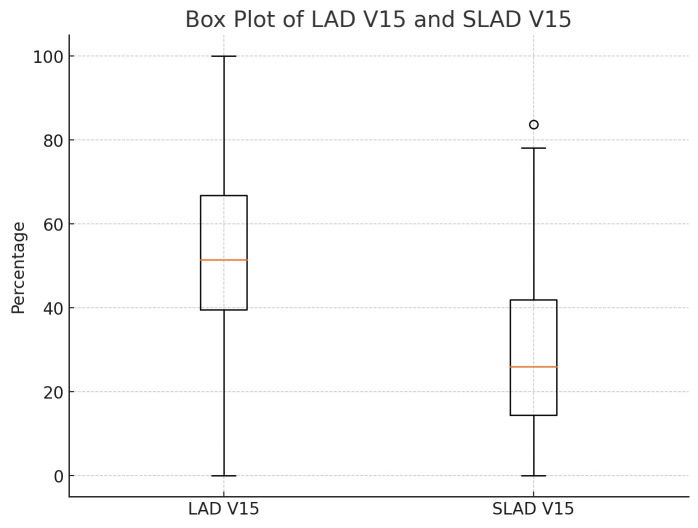
**Box plots of LAD V15 in standard and sparing planning techniques, shown side by side**. LAD V15, LAD volume receiving 15 Gy in standard plan; SLAD V15, LAD volume receiving 15 Gy in sparing plan; LAD, left anterior descending artery.

**Fig. 2.**
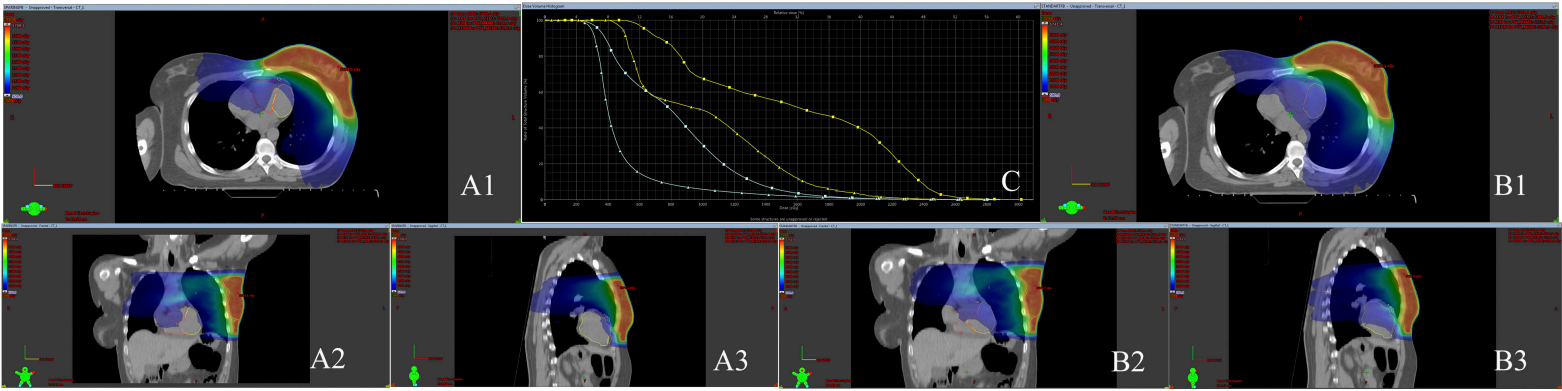
**Cross-sectional CT images of a patient with the LAD and LV delineated**. (A1,A2,A3) LAD/LV sparing plan transverse, coronal and sagittal sections. (B1,B2,B3) Standard plan transverse, coronal and sagittal sections. (C) DVH image of LAD and LV cardiac substructures in sparing and standard plans. Isodose line represents 5 Gy. Additionally, the dose-volume histogram (DVH) displays the dose distributions for the LAD (yellow) and LV (green). LAD, left anterior descending artery; LV, left ventricle; 3D, three-dimensional; PTV, planning target volume; cGy, centigray; Gy, gray; CT, computed tomography; ▲: standard plan LV dose distribution; ▲: standard plan LAD dose distribution; ■: sparing plan LV dose distribution; ■: sparing plan LAD dose distribution.

When evaluated individually, 26 patients with an initial LAD V15 dose greater than 50% experienced a reduction to 50% or below after VMAT plan re-optimization (Fig. 1). The number of patients with an LAD V15 dose greater than 50% in the standard plan was 40. Additionally, we successfully reduced the LAD mean dose from above 20 Gy to below 20 Gy in 13 patients. The number of patients with an LAD mean dose above 20 Gy in the standard plan was 16. Cross-sectional CT images of a patient whose mean LAD dose was reduced to below 20 Gy are shown in Fig. 2.

## 4. Discussion

In this study, we created new treatment plans by incorporating the LAD and LV organs into standard radiotherapy plans. We observed significant reductions in heart, LV, and LAD doses. For the other specified OARs, a significant increase in the mean dose to the right breast was observed. The clinical relevance of the mean contralateral breast dose is not well established in the current literature, especially compared to genetic and histological risk factors. Zurl *et al*. [14] stated that the increased mean contralateral breast dose associated with DIBH should not impact clinical decision-making regarding the excess risk of contralateral breast cancer (CBC). Current CBC risk assessment tools do not account for the dose received by the contralateral breast, and only ask whether radiotherapy was administered [15]. We also believe that a 3% increase in the contralateral breast dose during VMAT plan re-optimization should not affect clinical decision-making, especially considering the substantial 43% reduction in LAD V15.

Given the stronger association between the LAD and MACE compared to the MHD, the significant reduction in the LAD V15 dose suggests that protecting the LAD may be crucial in lowering the risk of MACE. Darby *et al*. [5] showed a significant elevation in the risk of ischemic heart disease after radiotherapy, and found a 7.4% increase in the risk of MACE per gray of MHD delivered to the patient. Our findings support the notion that this potential risk can be mitigated by protecting the LAD by including the OARs in treatment optimization. Similar dose reductions have been reported in the literature for patients using DIBH. Parlar *et al*. [16] observed a 50% reduction in MHD with DIBH compared with free-breathing techniques. Wolf *et al*. [17] also reported significant reductions in heart and LAD doses using the DIBH technique, highlighting its effectiveness in sparing cardiac structures. For patients unable to perform DIBH, the method developed in this study is a viable alternative to reduce the risk of MACE.

The use of intensity-modulated radiotherapy (IMRT) plans instead of three-dimensional conformal radiotherapy (3D-CRT) has been documented in the literature to reduce doses to the LAD and the heart. Garg and Kumar [18] found that IMRT plans significantly reduced the MHD and LAD by 30% and 25%, respectively, compared to 3D-CRT plans. The improved dose distribution offered by IMRT and VMAT techniques provide significant advantages for protecting critical organs. The literature reports that when the LAD dose exceeds 20 Gy, the risk of radiation-induced coronary stenosis increases by five-fold. Wennstig *et al*. [19] observed that patients receiving more than 20 Gy to the LAD had a significantly higher frequency of coronary artery interventions. In our study, we successfully reduced LAD doses to below 20 Gy in 13 out of 16 patients, thereby demonstrating the efficacy of our approach.

Van den Bogaard *et al*. [20] examined the impact of a LV V5 dose on MACE and found a significant risk reduction with lower LV V5 doses. Similar significant dose reductions were achieved in our patients. For instance, we observed a reduction in the LV V5 dose by 21% compared with conventional planning methods.

Arslan *et al*. [21] previously incorporated the LAD and LV into their optimization algorithm to minimize doses to these critical structures. Despite these efforts, the study’s limited sample size resulted in a statistically significant reduction in only D98, whereas the reduction in LV V5 was not statistically significant. Arslan *et al*. [21] reported a 12% reduction in the mean LAD dose and an 8% reduction in the mean LV dose in their cohort of 20 patients. Our study corroborates their findings. Using an improved study design with a larger sample size, we have established that this method can effectively lower doses to the LAD and LV. We achieved these results even when the differences between treatment plans were not statistically significant, highlighting the eficacy of our optimization algorithm.

Wang *et al*. [22] found that even low-dose radiation can cause perfusion damage in the irradiated areas. Therefore, adhering to the ALARA (As Low As Reasonably Achievable) principles, we aimed to minimize radiation doses to the lowest possible levels.

Radiotherapy-induced cardiotoxicity is a significant concern in the treatment of breast cancer. In a study by Díaz-Gavela *et al*. [23], the doses received by the heart during radiotherapy increased the risk of cardiac disease. The study revealed that the average heart and LAD doses were associated with increased cardiotoxicity. Specifically, higher doses to the LAD artery were associated with a greater risk of cardiac events. The maximum heart dose also plays a crucial role in determining this risk. These findings underscore the importance of minimizing doses using advanced radiotherapy techniques. Heart-sparing radiotherapy methods and multidisciplinary approaches are critical for reducing long-term cardiac complications and improving overall survival and quality of life for breast cancer patients [23].

Yeşildere and colleagues [24] conducted a dosimetric comparison between proton and photon therapies for the treatment of left-sided breast cancer. The study found that proton therapy significantly reduced doses to the heart and LAD. Specifically, proton therapy reduced the mean heart dose by 45% and the mean LAD dose by 50%. This dose reduction underscores the potential of proton therapy to minimize cardiac side effects. These findings suggest that proton therapy may be more advantageous for breast cancer treatment [24].

The limitations of this study include that only patients with left-sided breast cancer were included in this study. Therefore, the results are not applicable to right-sided breast cancer. Due to the low number of FB patients, comparative analysis of the sparing technique between DIBH and FB CT scans should be performed. This study was conducted retrospectively, which may introduce potential biases inherent to retrospective analyses, such as sampling bias and incomplete data collection.

## 5. Conclusions

Our method can be used with other advanced radiotherapy techniques to reduce the risk of MACE in patients with early-stage breast cancer. Future prospective studies should more comprehensively evaluate the effectiveness of LAD and LV protection strategies and their role in reducing MACE in clinical practice.

## Availability of Data and Materials

The datasets used and/or analyzed during the current study are available from the corresponding author on reasonable request.
